# Neuronal and Glial Biomarkers in Urine of Athletes with Different Risks of Head Trauma to Monitor Sports-Related Concussions

**DOI:** 10.1007/s12035-025-05507-y

**Published:** 2025-12-20

**Authors:** Konstantin Kohlhase, Valentin Vollmann, Ferdinand O. Bohmann, Christian Grefkes, Antonia Heuser, Klodiana Meci, Jens Göpfert, Christian Foerch

**Affiliations:** 1 Department of Neurology, Goethe University Frankfurt, University Hospital, Frankfurt Am Main, Germany; 2 Natural and Medical Sciences Institute, University of Tübingen, Reutlingen, Germany; 3 Department of Neurology, RKH Klinikum Ludwigsburg, Ludwigsburg, Germany

**Keywords:** Mild traumatic brain injury, MTBI, Brain trauma, Non-invasive, Point of care

## Abstract

Neuronal and glial biomarkers like glial fibrillary acidic protein (GFAP), ubiquitin C-terminal hydrolase-L1 (UCH-L1), tau protein, and neurofilament light chain (NfL) in serum are reported to be beneficial in detecting sports-related concussions (SRC) or intracranial trauma sequelae. However, routine blood measurements are invasive, and a non-invasive approach using urine could be advantageous. This prospective study analyzed urine samples from athletes with varying head trauma risks: high-risk (boxing), moderate-risk (American football, soccer), and low-risk (endurance sports). Samples were collected before (pre) and 48–72 h after (post) competition. Consecutive matches per athlete were sampled. Biomarker concentrations were adjusted for urine dilution using the creatinine ratio (CR). Statistical differences between groups were assessed using the Kruskal-Wallis test. A total of 48 athletes (boxing 11, American football 18, soccer 10, endurance 9) provided 112 samples. Ten SRC were recorded (boxing 9, soccer 1). Boxing athletes had the highest biomarker-CR, with significant differences in tau-CR and UCH-L1-CR compared to American football and endurance athletes (*p* = 0.005 and *p* = 0.001, respectively). No significant differences were found for NfL-CR or GFAP-CR. No significant changes were observed for biomarker-CRs 48–72 h after SRC. However, in a pooled analysis of all subsequent samples after a SRC, irrespective of the latency of sampling, there were significantly higher values for tau-CR and UCH-L1-CR (*p* = 0.02). Significantly higher levels of tau-CR and UCH-L1 were found in high-risk sports, potentially reflecting increased head impacts. However, an early increase of biomarker-CR within 72 h after SRC was not observed.

**Supplementary Information:**

The online version contains supplementary material available at 10.1007/s12035-025-05507-y.

## Introduction

Sports-related concussion (SRC) represents the mildest and most common form of sport-associated traumatic brain injuries (TBI), occurring at an average rate of 0.4–1.4 cases per 1000 athlete exposures [1, 2]. Particularly, contact sports such as American football, rugby, hockey, and combat sports are at an increased risk of SRC [3–5]. In this regard, SRC is defined as a direct blow to the head, neck, or body resulting in an impulsive force transmitted to the brain, with symptoms appearing immediately or within minutes to hours after the incident, typically resolving within a few days [6]. Over the past decade, SRC has received increasing attention in the monitoring of athletes due to the risk of persisting neurological symptoms after concussion, prolonged recovery, and the risk of chronic sequelae following repetitive SRC or subconcussive impacts [7–10]. While conventional neuroimaging is typically unremarkable in SRC, advanced MRI techniques such as quantitative and diffusion MRI may reveal subtle white matter abnormalities indicative of cerebral microtrauma [11–13]. However, the diagnosis and monitoring of SRC remain challenging, especially during the acute phase post-injury, with the potential of missing acute intracranial injuries [6]. In this context, blood-based neuronal and glial biomarkers such as glial fibrillary acidic protein (GFAP), ubiquitin C-terminal hydrolase-L1 (UCH-L1), tau protein, and neurofilament light chain (NfL) have been increasingly investigated for their roles in diagnosing SRC, predicting severe complications (e.g., intracranial hemorrhage), and estimating neurobiological recovery [14, 15]. Among these, GFAP and UCH-L1 have shown the most significant elevations during the acute phase after SRC and have demonstrated utility in predicting intracranial hemorrhages, whereas NfL concentrations have shown a delayed increase several days post-injury that was more correlated with the duration and prognosis of symptoms [15–17]. Based on this increasing evidence, the latest consensus recommendation of the ACRM (American Congress of Rehabilitation Medicine) has enabled the additional use of imaging or laboratory findings such as biomarkers for the diagnosis of SRC, but without providing specific details [18]. Besides acute diagnostics, neuronal and glial biomarkers show significant potential for monitoring brain injury in athletes with persistent symptoms or those participating in high-risk sports with frequent head impacts or subconcussive events [19, 20]. However, repetitive blood sampling is associated with logistical challenges and is often met with reluctance from athletes due to its invasiveness. In this regard, urine-based measurements offer a promising, non-invasive, and easily repeatable method that could be self-administered by athletes.

The aim of this study was to evaluate GFAP, NfL, UCH-L1, and tau protein in the urine of athletes exposed to different risks of head trauma. Specifically, we investigated whether early urine biomarker levels after head impact could predict SRC and whether biomarker expression differs between sports with varying risks of head injury.

## Material and Methods

This prospective single-center study was conducted between February 2022 and April 2023 at the Department of Neurology of the University Hospital Frankfurt, Goethe-University, Germany. The study protocol was approved by the Institutional Review Board of the Goethe University Frankfurt (IRB number 2021-442). Written informed consent was obtained from all participants before the samples were acquired. The study was aligned with the Standards for Reporting Diagnostic Accuracy (STARD) [21].

### Inclusion and Exclusion Criteria

Sports with different risks of head injuries were defined as follows: (I) high risk, combat sports such as boxing or mixed martial arts (MMA); (II) moderate risk, American football and soccer (note: Although American football is technically a high-risk sport for head injuries, it has been grouped as moderate due to the general helmet requirement.), (III) low risk, endurance sports (e.g., jogging, biking and swimming). As a rationale, we classified combat sports (e.g., boxing, MMA) as high risk as these disciplines often intend to induce loss of consciousness, with reported concussion rates up to 159 per 1000 athlete exposures (AEs) [5]. Collision and contact sports, such as American football and soccer, were categorized as moderate risk, as body contact is either intentional or highly likely during play, with concussion rates up to 3.02 and 1.38 per 1000 AEs, respectively [2]. However, given the differences between these sports, we also conducted separate analyses for each. Endurance sports (e.g., running, swimming, cycling) were classified as low risk, as they typically lack direct head impact or physical contact that would likely result in concussions.

*Inclusion criteria* for athletes with a moderate to high risk of sports-associated head trauma were as follows: (I) combat sports, American football or soccer with participation in training (sparring) or competitive fights (with or without head protection) and football or soccer matches, (II) age ≥ 18 years. Athletes with low risk of head trauma were included due to the following criteria: (I) performance of endurance sports (e.g., jogging, biking, and swimming), (II) no active participation in sports matching the criteria of moderate or high-risk sports. *Exclusion criteria* were as follows: (I) diagnosis of brain tumor, multiple sclerosis or neurodegenerative diseases, TBI with intracranial sequelae (e.g., hemorrhage, contusion) or previous ischemic/hemorrhagic stroke within the last 3 months.

### Sample Acquisition and Definition of mTBI

**Fig. 1:**
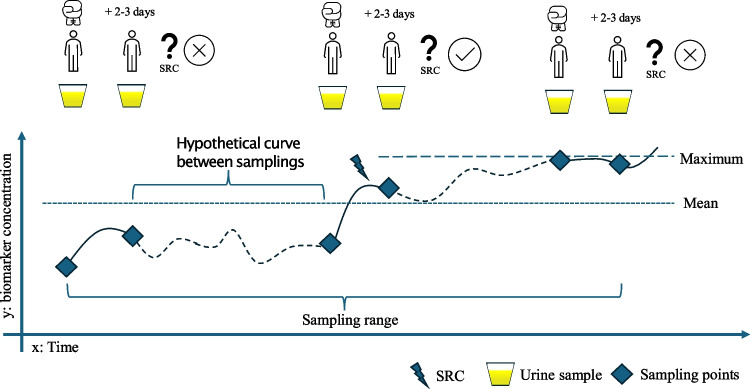
The sample collection procedure using a boxer as an example. The samples were taken the same day before (pre) a fight and 2–3 days (post) afterwards. A questionnaire was completed for each post-sample collection to determine whether a head trauma had knowingly occurred and whether symptoms suggestive of SRC had existed since then. For each athlete, further measurements could be taken during subsequent fights, allowing multiple measurement points for each individual. The period between the first and last sample collection was defined as the sampling range. In the chart, a hypothetical trajectory of the biomarker concentrations between two measurement points (pre and post), which was not covered, is depicted with a dotted line. The biomarker concentrations measured at the respective time points, before and after a match (with or without SRC), as well as the average concentration of all samples according to sport type, and the maximum concentration in the urine achieved per athlete, were statistically evaluated. Abbreviations: SRC, sports-related concussion

Sample acquisition in the high and moderate risk group was performed 48–72 h after a match or fight (“post-impact”) with a potential head impact. Due to the high probability of SRC or subconcussive head impacts in the high risk group, an additional “pre-impact” sample was obtained on the same day before the respective fight, when possible. This was optional for the moderate risk group due to practical reasons (e.g., admittance of most of the athletes directly to the match without time to acquire a pre-impact sample or post-impact contact of the athletes in case of a SRC without pre-impact sampling). The standard sample acquisition protocol and hypothetical course of biomarkers in urine for high-risk athletes are displayed in Fig. 1. During each post-impact sampling, all participants completed a questionnaire about the prior match or fight. The questionnaire included the following questions: Did the athlete experience head trauma (yes/no), how was the intensity of the trauma subjectively assessed on a numerical analog scale (1–10; low to severe), did unconsciousness or retrograde amnesia occur for the head trauma, and did neurological symptoms exist since then (somatic [e.g., headache, nausea or vomiting], cognitive impairment [e.g., disorientation, slowed reaction time, “feeling like in a fog”], gait unsteadiness, behavioral abnormality [e.g., irritability, emotional lability], or disturbance of sleep/wake rhythm [e.g., somnolence]). The report of head trauma in combination with at least one neurological symptom was considered as SRC and aligned with the *Consensus statement on concussion in sport—the 5th international conference on concussion in sport 2016* [22]. A reported head trauma without neurological symptoms was classified as subconcussive impact. During the collection period, athletes from high and moderate risk sports were able to provide multiple urine samples from subsequent matches or fights following the same procedure as described above. Urine from endurance athletes was collected one time, and the time of urine collection was not standardized in relation to exercise but was at a time convenient for the endurance athlete. The time interval between the first and last sample defined the individual sampling range per athlete. All urine samples (approx. 10 ml) were collected as spontaneous urine, pipetted into Eppendorf tubes after being centrifuged, and afterwards deep-frozen at −80 °C. The total time between urine collection and freezing was not allowed to exceed 6 h [23].

### Biomarker and Kidney Parameter Measurement

The measurement was performed using the Simoa® Human Neurology 4-Plex assay (N4PA, Quanterix, Billerica, MA, USA), an ultrasensitive immunoassay for the quantitative determination of NfL, t-tau, GFAP, and UCH-L1 in human serum, plasma, and cerebrospinal fluid (CSF). Since the assay was not validated by the manufacturer for use with urine samples, a pre-validation was conducted prior to sample analysis to demonstrate its suitability for analyzing urine, applying a fit-for-purpose approach. This validation involved defining the upper and lower limits of quantification (ULOQ and LLOQ) for each analyte, which were defined based on the highest and lowest calibration curve points provided by the manufacturer for the respective kit lot. Specifically, the quantification ranges (in-assay concentrations, not accounting for sample dilution) were as follows: t-tau 0.154–77.9 pg/mL, NfL 0.466–362.0 pg/mL, GFAP 1.0–815.0 pg/mL, and UCH-L1 13.2–11,889.0 pg/mL. In addition, assay precision and accuracy were evaluated using quality control (QC) samples. Intra-assay precision was below 23% coefficient of variation (CV) for all analytes, and inter-assay precision remained below 26.8% CV. Intra-assay accuracy ranged from 79.2% to 103%, and inter-assay accuracy from 92.7% to 114% for all analytes, as determined using both native and spiked urine samples to cover the assay ranges. More specifically, the QC samples were prepared by mixing different native urine samples with synthetic urine (Order No. 1005-D-50, Synthetic Urine e.K., Eberdingen-Nussdorf, Germany) in various proportions. To cover the full assay range, one of the samples was prepared entirely in synthetic urine and spiked with calibrator material from the kit. Additionally, recombinant UCH-L1 (Order No. 6007-CY-020, R&D Systems, Minneapolis, MN, USA) was added to extend the concentration range for UCH-L1 into the high end of the assay range. The nominal concentrations of the QC samples were determined prior to the validation and were used as reference values for the calculation of accuracy. Furthermore, sample parallelism was assessed, and the analytical stability of the samples stored at both refrigerated and room temperature was evaluated. Stability was confirmed for at least 24 h under both conditions for all analytes. Reproducibility was tested with nine native urine samples, with the CVs ranging from 0.66% to 32.2% across all samples and analytes. Based on dilution series, a dilution factor of 4 was selected for 109 of 112 samples. In 3 samples with a value below the LOQ/LOD, a remeasurement was carried out using a dilution factor of 2, which yielded values within the measuring range. The impact of freeze-thaw cycles on frozen samples was also evaluated, showing no effect on any analyte after at least two cycles. Based on these results, the assay was deemed suitable for use in the present study. The samples were analyzed in three independent runs conducted on three separate days, with each run including four levels of QC samples. Two of the three runs included duplicate sets of QC samples, while the third run included only a single set due to a limited number of total samples. Intra-assay coefficients of variation (CVs) for the QC samples in the two runs with duplicate QC sets ranged from 0.8% to 14.1% for Tau, 0.5% to 17.5% for NfL, 2.4% to 16.8% for GFAP, and 0.2% to 13.9% for UCH-L1. Inter-assay variation, assessed by the CVs of back-calculated QC concentrations across all three runs, ranged from 3.3% to 12.0% for Tau, 4.5% to 21.2% for NfL, 3.8% to 9.9% for GFAP, and 3.4% to 14.9% for UCH-L1 demonstrating acceptable inter-run variability for all four analytes.

Renal parameters in both urine and serum were analyzed using a fully automated photometric analyzer. Serum creatinine was measured with the Cobas 8000 c701 module, while urinary creatinine and albumin were analyzed using the Cobas 8000 c502 module (Roche Diagnostics GmbH, Mannheim, Germany). Serum albumin was quantified using an immunoturbidimetric assay (measurement range 0.3–40 mg/dL). Creatinine concentrations in serum (range 0.17–24.9 mg/dL) and urine (range 4.2–622 mg/dL) were determined using the Jaffé method [24, 25].

### Statistical Analysis

Statistical analysis was performed using IBM SPSS® (IBM Corp. Released 2021. IBM SPSS Statistics for Macintosh, Version 29.0.1.0 Armonk, NY: IBM Corp). The biomarker-creatinine-ratios (biomarker-CR = absolute biomarker concentration in urine [mg/dl]/creatinine in urine [mg/dl]) [26], albumin in urine, and albumin-creatinine-ratios (ACR) were evaluated for significant intergroup differences. As the dilution-corrected biomarker-CR is described to be more robust than the absolute urinary biomarker concentration, it was chosen as the primary value reported for biomarkers [26, 27]. Urine biomarker concentrations were corrected, if the value was below or above the limit of assay linearity. Measurements below the lower limit of quantification (LLOQ) of the assay were set at LLOQ/2 (pg/mL), while values below the limit of detection (LOD) of the assay were set at 0.00 (pg/mL). Values above the upper limit of quantification (ULOQ) were set at the assay’s ULOQ (pg/mL) (Supplementary Table 1). The normality of all parameters was assessed using the Kolmogorov-Smirnov test, and normal distribution was rejected for all variables. Therefore, non-parametric tests (Kruskal-Wallis test and Mann-Whitney-*U*-test) were used to determine intergroup differences and were reported as medians (first quartile–third quartile). For comparisons across more than two independent groups (e.g., across types of sports), a Kruskal-Wallis test was performed, followed by pairwise comparisons to assess intergroup differences in biomarker-CR in urine, age, urinary albumin, and ACR. Variables with categorical scale were evaluated using a chi-square test. With regard to SRC, a Mann-Whitney-*U* test was performed at a group level to compare biomarker-CR in samples with or without SRC collected within 72 h, as well as a pooled analysis of biomarker-CR in all samples collected after SRC regardless of the time point of sampling. Furthermore, the maximum biomarker-CR during the assessment period was calculated for each athlete (biomarker CR [Max]) and likewise analyzed for intergroup differences. The diagnostic accuracy of biomarkers to differentiate between high-risk sports (boxers) and other sports was expressed as area under the curve (AUC) and calculated using a ROC (receiver operating characteristic) analysis. *p*-values were corrected for multiple testing using the false discovery rate (FDR) adjusted *p*-value or Bonferroni correction. A *p*-value < 0.05 was considered significant.

## Results

### Baseline Characteristics

In total, 48 athletes provided a cumulative 112 samples (Table 1). In the high-risk group, 43 samples were obtained from 11 boxers (median [Q1–Q3]: 25.0 [21.0–27.0] years; 100% male), with a median sampling range of 49 days (maximum 254 days). The moderate-risk group consisted of 34 samples from 10 soccer players (22.0 [21.1–25.8] years, 100% male, median sampling range 7 days [max 93]) and 26 samples from 18 American football players (32.0 [25.5–37.0] years, 100% male, range 0 days [max 26]). A total of 9 samples were obtained from endurance athletes (24.0 [23.0–24.5] years; 100% male, median sampling range 0 days [maximum 0 days]), representing the low or no increased risk group. A total of 10 SRC from 8 different athletes were reported, 9 of which occurred in boxers and 1 in soccer players. The baseline characteristics as well as SRC-related symptoms are summarized in Table 1.

**Table 1:** Baseline characteristics, number of athletes and samples in relation to the practiced sport

Sport	Athletes [*n*=48]	Samples [*n*=112]	SRC [*n*=10]	Age [years]	Sex [%Male]	Sampling range [days]	Albumin in urine [mg/L]	ACR
Boxing	11	43	9	25.0 [21.0 – 27.0]	100%	Median: 49 Max: 254	3.0 [3.0 – 7.5]	2.7 [0.7 – 4.5]
Soccer	10	34	1	22.0 [21.1 – 25.8]	100%	Median: 7 Max: 93	5.0 [3.0 – 10.3]	3.4 [0.8 – 5.5]
American football	18	26	0	**32.0 [25.5 – 37.0]** *p* = 0.016	100%	Median: 0 Max: 26	3.0 [3.0 – 5.5]	2.0 [0.9 – 3.5]
Endurance sports	9	9	0	24.0 [23.0 – 24.5]	100%	-	3.0 [3.0 – 5.0]	1.0 [0.0 – 4.8]
Symptoms after SRC (*n* = 10)								
	Headache	Gait instability	Sleep Disturbance	Memory Loss	Cognitive	Behavior Disturbance	Loss of Consciousness	Intensity
***N***	10	0	2	2	3	1	1	5.7 [4.3 – 7.5]

The age and sex distribution, the time between first and last sample (“sampling range”), as well as kidney parameters (albumin in urine and ACR), were specified. Furthermore, the number of SRC, the corresponding symptoms as well as a self-reported graduation of the impact intensity using a numerical analogue scale (1 – 10, less to severe) are provided. Values are reportes as median [first – third quartile]. For the analysis of intergroup differences in age and kidney parameters, a Kruskal-Wallis test for numeric variables was used. A *p*-value < 0.05 was considered significant and corrected for multiple testing using a false discovery rate (FDR). Significant values are marked in bold

*ACR* albumin-creatinine-ratio; *SD* standard deviation; *SRC* sports-related concussion

### Biomarker Measurements in Urine

**Fig. 2:**
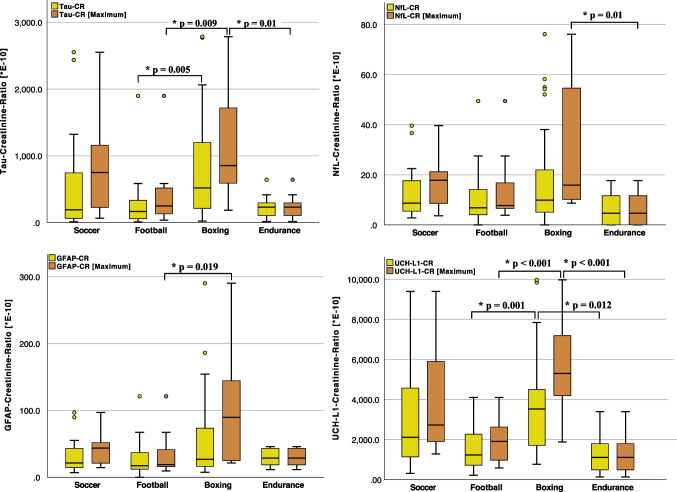
The box-plots for the biomarker-CR (scientific notation: biomarker-CR*E−10) of tau, NfL, GFAP, and UCH-L1 in urine in relation to the type of sport. The biomarker-CRs (yellow) as well as the maximum biomarker-CR per athlete (orange) are shown. A Kruskal-Wallis test with subsequent pairwise comparison was used to determine intergroup differences between sports. A *p*-value < 0.05 was considered statistically significant and depicted in the figure. Abbreviations: CR, creatinine ratio; GFAP, glial fibrillary acidic protein; NfL, neurofilament light chain; UCH-L1, ubiquitin carboxy-terminal hydrolase L1

The biomarker-CRs were examined for intergroup differences using a Kruskal-Wallis test with subsequent pairwise comparisons. Boxers showed the numerically highest values of biomarker-CRs compared to all other sports. These differences were significant for tau-CR (517.5 [205.5–1253.5] E^−10^) compared to American football players (166.7 [56.2–363.1] E^−10^ *p* = 0.005), as well as for UCH-L1-CR (3523.3 [1671.6–4695.7] E^−10^) compared to American football players (1235.3 [693.0–2302.1] E^−10^, *p* = 0.001) and endurance athletes (1110.1 [348.5–2298.2] E^−10^, *p* = 0.012). However, NfL-CR and GFAP-CR did not show significant intergroup differences across the different sports (Table 2). When considering the maximum biomarker-CR as a possible measure of the extent of neuronal or glial damage during the sampling period, for example, as an expression of previously noticed or unnoticed head trauma (Fig. 1), boxers showed significantly higher values for tau-CR (852.3 [516.5–1775.6] E^−10^) compared to endurance athletes (231.6 [73.5–355.6] E^−10^, *p* = 0.01) and American footballers (248.6 [122.0–518.5] E^−10^, *p* = 0.009), for NfL-CR (15.9 [9.5–55.0] E^−10^) compared to endurance athletes (4.7 [0.0–11.9] E^−10^, *p* = 0.01), for GFAP-CR (89.6 [23.3–154.2] E^−10^) compared to American footballers (18.8 [15.0–43.9] E^−10^, *p* = 0.019), and for UCH-L1-CR (5301.0 [3965.7–7490.0] E^−10^) compared to endurance athletes (1110.1 [348.5–2298.2] E^−10^, *p* < 0.001) and American footballers (1903.0 [922.0–2675.6] E^−10^, *p* < 0.001). Table 2 summarizes the measured biomarker-CRs and their corresponding statistical analyses. Biomarker-CRs are additionally illustrated in Fig. 2 using box-plots. The absolute urine concentrations of the investigated biomarkers are reported for completeness in Supplementary Table 2.

**Table 2:** The dilution-corrected biomarker-CR and the maximum biomarker-CR during the sampling range per athlete for tau, NfL, GFAP and UCH-L1 in urine in relation to the type of sport

**Biomarker**	**Soccer** **[** ***n*** **=34]**	**American Football** **[** ***n*** **=26]**	**Boxing** **[** ***n*** **=42]**	**Endurance** **[** ***n*** **=9]**	***p*** **-value**
Tau-CR*E^-10^	191.0 [58.0 – 745.9]	166.7 [56.2 – 363.1]	**517.5** [205.5 – 1253.5]*	231.6 [73.5 – 355.6]	* = 0.005 [vs. football]
NfL-CR*E^-10^	8.7 [5.4 – 17.7]	6.8 [4.0 – 14.4]	9.9 [4.9 – 23.2]	4.7 [0.0 – 11.9]	> 0.05
GFAP-CR*E^-10^	21.3 [14.1 – 43.2]	17.2 [11.7 – 37.8]	26.7 [16.0 – 76.3]	28.5 [15.1 – 43.6]	> 0.05
UCH-L1-CR*E^-10^	2114.4 [1104.5 – 4571.9]	1235.3 [693.0 – 2302.1]	**3523.3** [1671.6 – 4695.7]*	1110.1 [348.5 – 2298.2]	* = 0.001 [vs. football), 0.012 [vs. endurance]
**Biomarker** **[Maximum]**	**Soccer** [*n*=10]	**American Football** *n*=18]	**Boxing** [*n*=11]	**Endurance** [*n*=9]	***p*** **-value**
Tau-CR*E^-10^	751.6 [208.6 – 1478.8]	248.6 [122.0 – 518.5]	**852.3** [516.5 – 1775.6] *	231.6 [73.5 – 355.6]	* = 0.01 [vs. endurance], 0.009 [vs. football]
NfL-CR*E^-10^	17.8 [7.9 – 21.5]	7.7 [6.1 – 18.0]	**15.9** [9.5 – 55.0]*	4.7 [0.0 – 11.9]	* = 0.01 [vs. endurance]
GFAP-CR*E^-10^	43.6 [20.5 – 52.4]	18.8 [15.0 – 43.9]	**89.6** [23.3 – 154.2]*	28.5 [15.1 – 43.6]	* = 0.019 [vs. football]
UCH-L1-CR*E^-10^	3821.5 [1864.5 – 6205.5]	1903.0 [922.0 – 2675.6]	**5301.0** [3965.7 – 7490.0]*	1110.1 [348.5 – 2298.2]	* < 0.001 [vs. endurance and football]

The values are presented as median (first quartile – third quartile) with a scientific notation given as the value*E^-10^. One biomarker-CR sample was not calculated in the boxing group due to a missing creatinine value in urine. For statistical analysis, a Kruskal-Wallis test was performed to determine intergroup differences between sports with a subsequent pairwise comparison. The *p*-value was corrected for multiple testing using a Bonferroni correction. A *p*-value < 0.05 was considered statistically significant and are marked in bold with the respective p-value for significant pairwise comparisons (*)

*CR* creatinine-ratio; *GFAP* glial fibrillary acidic protein; *NfL* neurofilament light chain; *UCH-L1* ubiquitin carboxy-terminal hydrolase L1

### Sports-Related Concussions

**Fig. 3:**
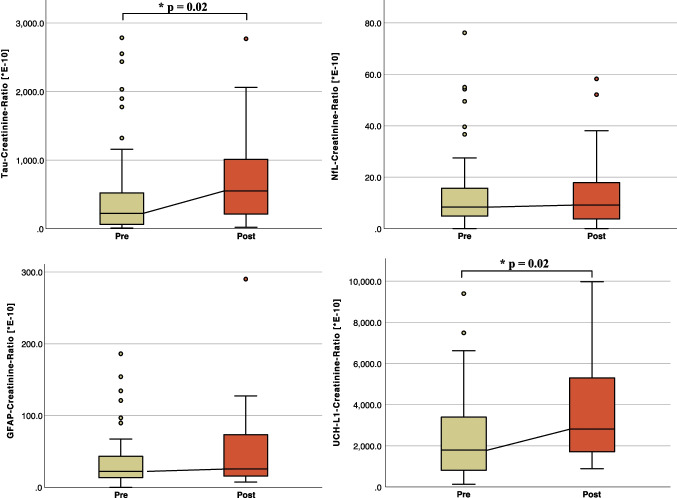
The box-plots for tau, NfL, GFAP, and UCH-L1 measured from urine for samples before (pre) and all subsequent samples after a SRC (post) regardless of sampling time. A Mann-Whitney-U test was used to determine intergroup differences between sports. A p-value < 0.05 was considered statistically significant and was indicated above. Abbreviations: CR, creatinine ratio; GFAP, glial fibrillary acidic protein; NfL, neurofilament light chain; SRC, sports-related concussion; UCH-L1, ubiquitin carboxy-terminal hydrolase L1

Urine samples of athletes were grouped into those obtained within 48–72 h after SRC compared to (I) the individual samples acquired before the fight or match (“pre-impact”) or (II) versus all samples irrespective of head trauma. Furthermore, an analysis was performed for all head traumas (SRC and subconcussion) versus all other samples. A pre- and post-impact sample pair was available in 6 of 10 SRC cases. No significant differences in biomarker-CRs were observed between these groups (Table 3 (A, B, and C)). In a post hoc analysis, all available samples from each athlete, before and after SRC, were pooled for comparison. This analysis revealed significantly higher values for tau-CR (629.4 [257.7 – 1572.0] E^−10^ vs. 229.6 [74.2 – 560.0] E^−10^, *p* = 0.02) and UCH-L1-CR (3506.1 [1920.2 – 4897.5] E^−10^ vs. 1791.3 [970.1 – 3500.0] E^−10^, *p* = 0.02) in post-SRC samples, regardless of the time interval between the SRC and sample collection. No significant differences were observed for NfL-CR and GFAP-CR (Table 3 (D), Fig. 3). The individual course of biomarker-CRs for athletes with concussion is visualized in Supplementary Fig. 1.

**Table 3:** Biomarker-CR for tau, NfL, GFAP and UCH-L1 in urine in regard to the occurrence of SRC or non-concussive head impact

**A**	**No SRC,** ***n*** **= 101**	**SRC,** ***n*** **= 10**	***p*** **-value**
Tau- CR*E^-10^	258.2 [94.9 – 688.4]	480.4 [138.8 – 702.0]	0.72
NfL- CR*E^-10^	8.0 [4.6 – 16.3]	12.8 [5.7 – 18.9]	0.72
GFAP- CR*E^-10^	23.3 [13.5 – 44.8]	24.5 [15.6 – 54.2]	0.74
UCHL1- CR*E^-10^	2031.8 [1094.9 – 3695.8]	2385.7 [1275.5 – 4741.0]	0.72
**B**	**Pre SRC (< 24h),** ***n*** **= 6**	**Post SRC (48–72h),** ***n*** **= 6**	***p*** **-value**
Tau- CR*E^-10^	554.4 [202.9 – 1937.1]	572.9 [303.2 – 983.4]	1.0
NfL- CR*E^-10^	12.1 [7.9 – 40.9]	16.6 [5.7 – 26.0]	1.0
GFAP- CR*E^-10^	32.0 [19.3 – 122.1]	34.7 [14.0 – 64.7]	1.0
UCHL1- CR*E^-10^	2087.4 [1404.4 – 4670.8]	2737.0 [1672.6 – 4741.0]	1.0
**C**	**No head impact,** ***n*** **= 92**	**Head impact [all],** ***n*** **= 19**	***p*** **-value**
Tau- CR*E^-10^	254.2 [80.5 – 659.3]	396.5 [125.0 – 743.5]	0.87
NfL- CR*E^-10^	8.4 [4.9 – 16.5]	9.2 [3.9 – 17.9]	0.87
GFAP- CR*E^-10^	23.2 [13.8 – 43.8]	25.7 [14.8 – 55.3]	0.87
UCHL1- CR*E^-10^	2036.1 [979.7 – 3612.2]	1956.4 [1354.5 – 5301.0]	0.76
**D**	**Pre SRC (pooled),** ***n*** **= 90**	**Post SRC (pooled),** ***n*** **= 21**	***p*** **-value**
Tau- CR*E^-10^	229.6 [74.2 – 560.0]	**629.4 [257.7 – 1572.0]**	**0.02**
NfL- CR*E^-10^	8.1 [4.9 – 16.1]	10.9 [3.2 – 17.5]	0.85
GFAP- CR*E^-10^	22.1 [13.1 – 43.4]	32.3 [19.0 – 68.8]	0.09
UCHL1- CR*E^-10^	1791.3 [970.1 – 3500.0]	**3506.1 [1920.2 – 4897.5]**	**0.02**

The biomarker-CRs were grouped according to the following categories: A –SRC with post-SRC sample acquisition 48–72h compared to non-SRC samples; B SRC with post-SRC sample acquisition 48–72h compared to the respective pre-SRC samples acquired within 24 hours before SRC – C: General raumatic head impact, including SRC and subconcussions with sample acquisition within 48-72h after impact; D – Composite group of all samples per athlete before [pre SRC] and after an SRC [post SRC] independent of sampling time; A Mann-Whitney-U-test was used to calculate the intergroup differences of the biomarker-CR between the subgroups. A *p*-value < 0.05 was considered statistically significant and corrected for multiple testing using a false discovery ratio (FDR), significant values are marked in bold. The values are presented as median (first quartile – third quartile) with a scientific notation given as the value*E^-10^

*CR* creatinine-ratio; *GFAP* glial fibrillary acidic protein; *NfL* neurofilament light chain; *SRC* sports-related concussion; *UCH-L1* ubiquitin carboxy-terminal hydrolase L1

### ROC Analysis

**Fig. 4:**
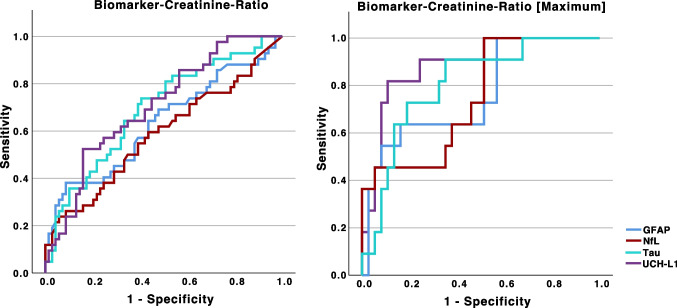
ROC (receiver operating characteristics) analysis for the prediction of whether a sample can be identified as belonging to sports with a high risk of traumatic head impacts (e.g., boxers) versus others sports with moderate to low risk using the biomarker-CR and the maximum measured biomarker-CR (maximum). Abbreviations: CR, creatinine ratio; GFAP, glial fibrillary acidic protein; NfL, neurofilament light chain; UCH-L1, ubiquitin carboxy-terminal hydrolase L1

A ROC analysis was performed to determine the predictive value of the biomarker-CR and biomarker-CR (maximum) in differentiating a sample from high-risk athletes against other sports with moderate to low risk of head injuries. In the ROC analysis of biomarker-CRs, the following AUCs were calculated: tau-CR of 0.69 (0.59–0.79; *p* < 0.001), NfL-CR of 0.59 (0.47–0.70; *p* = 0.14), GFAP-CR of 0.63 (0.52–0.74; *p* = 0.02) and UCH-L1-CR of 0.71 (0.61–0.80; *p* < 0.001). When the maximum biomarker CR was used, the respective AUC was as follows: tau-CR (Max) of 0.81 (0.69–0.94; *p* < 0.001), NfL-CR (Max) of 0.75 (0.59–0.91; *p* = 0.002), GFAP-CR (Max) of 0.76 (0.60–0.93; *p* = 0.002), and UCH-L1-CR (Max) of 0.89 (0.78–0.99; *p* < 0.001) (Fig. 4).

## Discussion

In this study, we observed significantly higher UCH-L1-CR and tau-CR in the urine of athletes with a high risk for repetitive head trauma and SRC compared to those with moderate or low risk. However, there was no significant increase in urine biomarker-CR after SRC, when samples were acquired within 48–72 h after impact.

These findings contrasted with previous blood-based studies. For example, a study from the University of Florida reported an increase in GFAP, t-tau, and NfL in blood within 48 h after SRC, with the highest diagnostic value for GFAP and NfL combined [14]. Similar results were reported in an analysis from the National Collegiate Athletic Association (NCAA) and the US Department of Defense Concussion, Assessment, Research and Education (CARE) Consortium, which showed elevated levels of GFAP, t-tau, and UCH-L1 within 24–48 h post-trauma, whereas NfL increased with a delay of 7 days after injury [15]. Another Canadian study demonstrated an increase in GFAP, NfL, and UCH-L1 during the first 3 days after SRC, with elevated levels persisting up to 28 days [28]. Although the studies generally showed a predictable increase in biomarkers within the first 72 h after SRC, the magnitude of increase was heterogeneous. Applied to our study, the absence of an increase in urinary biomarkers within 48–72 h could be attributed to limited protein penetration into urine, as well as differences in kinetics between urine and blood [29]. In this regard, a previous study from our research group reported that the urinary excretion of biomarkers was increased in the presence of glomerular barrier impairment, particularly for GFAP and, to a lesser extent, for NfL [30, 31]. Due to the low ACR in the investigated athletes, which indicates an intact glomerular barrier function, the transmission of proteins into urine may have therefore been physiologically restricted [32]. While data about the kinetics of blood-urine release for the evaluated biomarkers is limited, studies on S100B after TBI suggest a slightly slower increase in urine compared to blood [33, 34]. A more frequent and longitudinal measurement, coupled with a urine-to-serum correlation, would be necessary to clarify this issue. However, as serum samples were not collected in the present study, no information is available about this aspect.

While it was not possible to demonstrate an increase in urine biomarker-CR within 48–72 h after SRC, there was a significant difference for tau-CR and UCH-L1-CR in high-risk athletes in a pooled analysis of samples after SRC (Table 3 (D) and Fig. 3) compared to those acquired before a documented SRC. A possible explanation could be that the trauma had induced an increase in serum values, but the penetration into urine was delayed [28]. Furthermore, subclinical concussions or unnoticed head impacts in the meantime could have additionally contributed to this increase [35, 36]. The difference in biomarker-CRs across high-risk sports was further accentuated when the highest measured value within the sampling range was considered, regardless of whether a SRC had been previously documented. However, since a systematic assessment of head impacts or SRC between two independent measurements has not been performed, those results are preliminary and should be re-confirmed in follow-up studies. Based on these suggestions, the value of urinary biomarker measurements could shift from a peri-traumatic measurement to a more longitudinal one, such as monitoring throughout a season in high-risk sports. The biomarker measurements could be further enhanced using radiological biomarkers (e.g., high-resolution MRI) and be correlated with the number of impacts and SRCs, as well as the time to symptom relief or persisting clinical complaints [28, 37]. In this context, it is of interest to evaluate whether elevated biomarker levels correlate with chronic sequelae following repetitive head trauma, e.g., whether tau protein levels correlate with the later onset of CTE, which is histopathologically characterized by the intracerebral deposition of tau protein [38].

An interesting observation was that American football players showed notably lower concentrations, although they were originally examples of a particularly high-risk group for head trauma [39]. This may be a result of the increasing awareness of chronic injuries such as CTE with now stricter regulations in American football, e.g., a general helmet requirement, higher helmet standards, or prohibition of head-tackles [40, 41]. In line with this, no SRCs were reported in American football and may also reflect an example of successful prevention. However, the results in American football players are limited by significant age differences as a positive correlation between serum GFAP concentration and age in adults has been described [42].

The highest difference in biomarker-CR was observed for UCH-L1 and tau in high-risk athletes. While both biomarkers are highly expressed in brain tissue, they are not completely brain-specific and were also found in non-neuronal tissues [43, 44]. Here, UCH-L1 was also reported to be expressed in the distal tubule, parietal epithelial cells, and podocytes of the kidney [45–47]. It was shown that UCH-L1 as a component of the ubiquitin-proteasome pathway accumulates in podocytes and parietal epithelial cells, particularly in the presence of acute glomerular damage (e.g., glomerulonephritides) [48–51]. The athletes included in the study neither had a known kidney disease, nor was there an absolute increase or relevant group differences in ACR as an indicator of glomerular kidney impairment [52, 53]. However, it cannot be ruled out that UCH-L1 detected in urine partly originated directly from the kidney, although this does not adequately explain the differences between athletes. Besides UCH-L1, there are also a few reports, partly from mouse models, of a moderate expression of tau protein in the proximal tubule of the kidney [43]. However, since the data on tau is more limited compared to UCH-L1, the significance of a renal origin of tau cannot be assessed yet. For future studies, it should be considered whether our results can be re-confirmed using phosphorylated tau isoforms, such as pTau 181, which are more specific to brain tissue and were found to be elevated after mild TBIs [54]. For GFAP and NfL there are, to our knowledge, no corresponding reports of a relevant occurrence in the kidney [55, 56].

## Limitations

This study contains limitations, which are described in the following: First, the small number of SRC reduces statistical power and may have obscured significant effects. Furthermore, pre-impact urine samples (< 24 h before SRC) were available for only 6 out of 10 SRC cases. This was primarily due to the identification of a potential SRC after the fight, based on athlete reports, thus precluding pre-impact measurement. Therefore, in addition to analyses of the pre- and post-impact pairs, we compared post-impact SRC samples to the remaining non-SRC samples. However, this comparison is limited by the fact that included athletes, especially boxers, who comprised the majority, are intrinsically at high risk of subconcussions and unnoticed head impacts, which could shift baseline biomarker levels. Supplementary Fig. 1 illustrates individual biomarker-CR trajectories, where UCH-L1, as the most significant marker across groups, showed an increase post-SRC in some athletes, including one case with repeated SRCs within a short interval. While these values showed a distinctive intra-individual increase, they appeared only marginally elevated in inter-individual comparison. This supported the hypothesis that intra-individual biomarker-CR trends may be more informative than absolute biomarker-CR values, although further research is needed to confirm this. Second, there was a difference in the sampling range between sports, so that boxers in particular were monitored over a longer period, while endurance athletes only had a single measurement. In this context, the samples were obtained at individual competitions instead of fixed intervals. In future projects, measurements should be extended by fixed intervals and additional consecutive measurements after SRC, also including sports without relevant risk of head trauma. Third, our study included only male athletes, limiting generalizability. When studying female athletes, special consideration must be given to the potential influence of the menstrual cycle, as biomarkers from menstrual blood loss may pass into urine, which could increase variability or deviations of biomarker concentrations. Finally, the assays used were not manufacturer-validated for urine. We performed preliminary validations to assess assay suitability, but NfL in particular showed imprecision at low concentrations. Both NfL and GFAP had a high proportion of values below the LLOQ, which we corrected using LLOQ/2. Although this is an accepted method, the high rate of imputed values may affect results and warrants cautious interpretation. Tau and UCH-L1 measurements were more reliable, with fewer values below LLOQ (see Supplementary Table 1).

## Conclusion

This study revealed significantly higher average values of UCH-L1-CR and tau-CR in urine of athletes with a high risk of head trauma compared to moderate and low risk sports. Although there was no increase in biomarker-CR within 48–72 h after SRC, there were significantly higher UCH-L1-CR and tau-CR in athletes who experienced a SRC during the sampling period.

## Supplementary Information

Below is the link to the electronic supplementary material.

ESM1 (DOCX 111 KB)

## Data Availability

The datasets are not publicly available due to German law restrictions but can be requested in anonymized format for the purpose of data reproducibility from the corresponding author on reasonable request.
