# Supplementary material for: Neuronal and Glial Biomarkers in Urine of Athletes with Different Risks of Head Trauma to Monitor Sports-Related Concussions
Source: Mol Neurobiol. 2025 Dec 20;63(1):314. doi: 10.1007/s12035-025-05507-y (PMC12717207; doi:10.1007/s12035-025-05507-y)
Supplement: Supplementary file 1 — (DOCX 111 KB) [file 12035_2025_5507_MOESM1_ESM.docx]

**Supplements:**

|  |  | **Boxing n=43** | **Soccer n=34** | **Football n=26** | **Endurance**  **n=18** |
| --- | --- | --- | --- | --- | --- |
| **Tau** | Within range | 86.0 % (n=37) | 97.1 % (n=33) | 100.0 % (n=26) | 83.3 % (n=15) |
|  | < LOD | 0.0 % (n=0) | 0.0 % (n=0) | 0.0 % (n=0) | 0.0 % (n=0) |
|  | < LLOQ | 0.0 % (n=0) | 0.0 % (n=0) | 0.0 % (n=0) | 0.0 % (n=0) |
|  | > ULOQ | 14.0 % (n=6) | 2.9 % (n=1) | 0.0 % (n=0) | 16.7 % (n=3) |
|  | No valid result | 0.0 % (n=0) | 0.0 % (n=0) | 0.0 % (n=0) | 0.0 % (n=0) |
| **NfL** | Within range | 23.3 % (n=10) | 2.9 % (n=1) | 0.0 % (n=0) | 16.7 % (n=3) |
|  | < LOD | 11.6 % (n=5) | 0.0 % (n=0) | 15.4 % (n=4) | 33.3 % (n=6) |
|  | < LLOQ | 65.1 % (n=28) | 94.1 % (n=32) | 84.6 % (n=22) | 50.0 % (n=9) |
|  | > ULOQ | 0.0 % (n=0) | 0.0 % (n=0) | 0.0 % (n=0) | 0.0 % (n=0) |
|  | No valid result | 0.0 % (n=0) | 2.9 % (n=1) | 0.0 % (n=0) | 0.0 % (n=0) |
| **GFAP** | Within range | 18.6 % (n=8) | 5.9 % (n=2) | 0.0 % (n=0) | 22.2 % (n=4) |
|  | < LOD | 0.0 % (n=0) | 0.0 % (n=0) | 3.8 % (n=1) | 0.0 % (n=0) |
|  | < LLOQ | 81.4 % (n=35) | 94.1 % (n=32) | 96.2 % (n=25) | 77.8 % (n=14) |
|  | > ULOQ | 0.0 % (n=0) | 0.0 % (n=0) | 0.0 % (n=0) | 0.0 % (n=0) |
|  | No valid result | 0.0 % (n=0) | 0.0 % (n=0) | 0.0 % (n=0) | 0.0 % (n=0) |
| **UCH-L1** | Within range | 95.3 % (n=41) | 85.3 % (n=29) | 92.3 % (n=24) | 77.8 % (n=14) |
|  | < LOD | 0.0 % (n=0) | 0.0 % (n=0) | 0.0 % (n=0) | 0.0 % (n=0) |
|  | < LLOQ | 4.7 % (n=2) | 14.7 % (n=5) | 7.7 % (n=2) | 22.2 % (n=4) |
|  | > ULOQ | 0.0 % (n=0) | 0.0 % (n=0) | 0.0 % (n=0) | 0.0 % (n=0) |
|  | No valid result | 0.0 % (n=0) | 0.0 % (n=0) | 0.0 % (n=0) | 0.0 % (n=0) |

**Supplementary Table 1:** The measured biomarker concentrations of tau, NfL, GFAP and UCH-L1 are summarized in relation to the assay thresholds divided into different sports (boxing, soccer, football, endurance). The limit ranges were determined using the calibration curves of the assay (concentration of lowest or highest calibrator concentration). Abbreviations: GFAP – *glial fibrillary acidic protein*, LOD – *limit of detection*, LLOQ – *lower limit of quantification*, NfL – *neurofilament light chain*, UCH-L1 - *ubiquitin carboxy-terminal hydrolase L1*, ULOQ – *upper limit of quantification*.

| **Biomarker** | **Soccer** [n=34] | **American Football**  [n=26] | **Boxing**  [n=43] | **Endurance**  [n=9] | **p-value (corrected: Bonferroni)** |
| --- | --- | --- | --- | --- | --- |
| Tau [pg/mL] | 32.2 [3.8 – 111.6] | 13.3 [5.9 – 47.0] | 68.8 [13.9 – 159.8] | 14.5 [9.7 – 39.2] | > 0.05 |
| NfL[pg/mL] | **1.0 [1.0 – 1.0]*** | 1.0 [1.0 – 1.0] | **1.0 [1.0 – 1.0]**** | 1.0 [0.0 – 1.0] | * = 0.034 [vs. endurance]  ** = 0.006 [vs. endurance] |
| GFAP [pg/mL] | 2.4 [2.4 – 2.4] | 2.4 [2.4 – 2.4] | **2.4 [2.4 – 2.4]*** | 2.4 [2.4 – 2.4] | * = 0.018 [vs. football] |
| UCH-L1[pg/mL] | 257.1 [78.9 – 755.6] | 109.3 [74.6 – 255.3] | **302.1 [169.5 – 541.7]*** | 95.2 [27.0 – 236.7] | * = 0.033 [vs. football],  0.049 [vs. endurance] |
|  | | | | | |
| **Biomarker [Maximum]** | **Soccer** [n=10] | **American Football**  [n=18] | **Boxing**  [n=11] | **Endurance**  [n=9] | p-value |
| Tau [pg/mL] | 126.6 [51.2 – 284.5] | 28.9 [8.5 – 63.8] | **117.8 [79.0 – 385.0]*** | 14.5 [9.7 – 39.2] | * = 0.024 [vs. endurance],  0.046 [vs. football] |
| NfL [pg/mL] | 1.0 [1.0 – 1.0] | 1.0 [1.0 – 1.0] | **1.0 [1.0 – 3.1] *** | 1.0 [0.0 – 1.0] | * < 0.001 [vs. endurance] |
| GFAP [pg/mL] | 2.4 [2.4 – 2.4] | 2.4 [2.4 – 2.4] | 2.4 [2.4 – 8.7] | 2.4 [2.4 – 2.4] | > 0.05 |
| UCH-L1 [pg/mL] | **424.6 [156.6 – 1283.5] *** | 124.7 [99.3 – 347.9] | **622.6 [301.4 – 1023.8]**** | 95.2 [27.0 – 236.7] | * = 0.042 [vs. endurance]  ** = 0.008[vs. endurance] |

**Supplementary Table 2:** The absolute biomarker concentration in urine (upper half) and the maximum concentrations during the sampling range (lower half) per athlete for tau, NfL, GFAP and UCH-L1 in urine in relation to the type of sport are presented. The biomarker concentrations are reported as median (First Quartile – Third Quartile). For statistical analysis, a Kruskal-Wallis was performed to determine intergroup differences of biomarker concentrations between sports with a subsequent pairwise comparison of the sports against each other. A p-value < 0.05 was considered statistically significant. The p-value was corrected for multiple testing using a Bonferroni correction. Significant values are marked in bold with the respective p-value for the significant pairwise comparisons of sports (*, **). **Abbreviations:** *GFAP - glial fibrillary acidic protein, NfL - neurofilament light chain, UCH-*L1 - *ubiquitin carboxy-terminal hydrolase L1*

**Supplementary Figure 1:**

**Supplementary Figure 1:** The figure illustrates the temporal progression of biomarker-to-creatinine ratios (CR) for tau, NfL, GFAP, and UCH-L1 in athletes with a confirmed sport-related concussion (SRC). The x-axis represents time in days, while the y-axis displays the respective biomarker-to-creatinine ratios. All samples collected within 48 to 72 hours post-SRC are specifically highlighted. To compare biomarker levels in samples taken before and after an SRC, a Mann-Whitney U test was performed (significance level: 0.05), which revealed no statistically significant differences. Athlete 10 exhibits a particularly noteworthy trajectory, having sustained three SRCs within a short timeframe (Day 0, Day 3, and Day 20). Notably, UCH-L1-CR shows a progressive increase following each SRC in this athlete. Additionally, Athletes 38 and 52 display an increase in UCH-L1-CR between the day before a match and 48–72 hours afterward. For Athlete 35, only a single post-SRC measurement was available, which showed a marked decrease in UCH-L1-CR by Day 12.
